# MRI-Based Meningioma Firmness Classification Using an Adversarial Feature Learning Approach

**DOI:** 10.3390/s25051397

**Published:** 2025-02-25

**Authors:** Miada Murad, Ameur Touir, Mohamed Maher Ben Ismail

**Affiliations:** Computer Science Department, College of Computer and Information Sciences, King Saud University, Riyadh 11451, Saudi Arabia; touir@ksu.edu.sa (A.T.); mbenismail@ksu.edu.sa (M.M.B.I.)

**Keywords:** meningioma firmness detection, deep learning, magnetic resonance images, convolutional neural network

## Abstract

The firmness of meningiomas is a critical factor that impacts the surgical approach recommended for patients. The conventional approaches that couple image processing techniques with radiologists’ visual assessments of magnetic resonance imaging (MRI) proved to be time-consuming and subjective to the physician’s judgment. Recently, machine learning-based methods have emerged to classify MRI instances into firm or soft categories. Typically, such solutions rely on hand-crafted attributes and/or feature engineering techniques to encode the visual content of patient MRIs. This research introduces a novel adversarial feature learning approach to tackle meningioma firmness classification. Specifically, we present two key contributions: (i) an unsupervised feature extraction approach utilizing the Bidirectional Generative Adversarial Network (BiGAN) and (ii) a depth-wise separable deep learning model were designed to map the relevant MRI features with the predefined meningioma firmness classes. The experiments demonstrated that associating the BiGAN encoder, for unsupervised feature extraction, with a depth-wise separable deep learning model enhances the classification performance. Moreover, the proposed pre-trained BiGAN encoder-based model outperformed relevant state-of-the-art methods in meningioma firmness classification. It achieved an accuracy of 94.7% and a weighted F1-score of 95.0%. This showcases the proposed model’s ability to extract discriminative features and accurately classify meningioma consistency.

## 1. Introduction

Cancer is the second leading cause of death globally, claiming over 9.6 million lives across 185 countries [1]. This alarming mortality rate is projected to reach 21.4 million deaths by 2030 [1]. The incidence and mortality rate of cancer in Saudi Arabia has witnessed a drastic increase, rising from 5% to 12% between 1990 and 2016. In fact, a brain tumor is defined as an abnormal growth or mass of cells that is typically categorized as either benign or malignant [2]. In particular, malignant brain tumors are among cancers associated with high death rates. They critically affect the patient cognitive capabilities and the quality of life [3]. In addition, it is hard for an expert radiologist to detect brain tumors at an early stage. This triggered the need for computer-aided detection (CAD) systems intended to reduce the time, the subjectivity, and the cost of such diagnoses [4].

Surgical removal represents the most prevalent therapy to overcome meningiomas [5]. In fact, meningiomas develop in the meninges that wrap the brain and spinal cord. It should be noted that benign tumors have the potential to grow larger, harm the brain, and result in life-threatening health issues. Approximately 90% of meningiomas are identified as benign tumors. The remaining 10% are typically malignant [3,6]. Meningiomas’ consistency, which varies from soft to firm, is a primary criterion for the surgical approach and the patient care to be prescribed by the healthcare provider. In particular, tissue suction is practiced to remove soft meningiomas. Conversely, removing firm meningiomas requires an invasive procedure [5].

The manual segmentation and analysis of brain tumor structural MRIs is a challenging and time-consuming task that can only be carried out by expert neuroradiologists [7]. In addition, the need for effective early detection of tumors has promoted the development of CAD systems intended to reduce the diagnosis time, subjectivity, and cost [4]. Utilizing CAD systems with early tumor detection capabilities would significantly improve the brain tumor identification as well as the corresponding treatment processes. Particularly, this would enable more appropriate and on-time treatment procedures for patients [8,9,10]. Conventional CAD systems rely on manually engineered features that aim to better represent the visual characteristics of benign and malignant cases [11,12,13,14]. In contrast, deep learning-based approaches relax this constraint as they determine automatically the relevant features without expert supervision.

Several deep learning-based approaches have been developed for the diagnosis and categorization of brain tumors [15]. In particular, CNN-based classification systems that do not require effective prior segmentation and selection of tumor areas were introduced [15,16]. In particular, transfer learning was exploited in [16] to solve a three-class brain tumor classification problem. The authors presented a classification system that uses the pre-trained GoogLeNet [17] to extract relevant features from brain MRI images. The researchers in [15] proposed a CNN architecture that associates a max-pooling layer with each convolutional layer, followed by flattening layers, and eventually a full connection. The resulting CNN model was trained using 3064 T-1 weighted CE-MRI images publicly available in [18]. Similarly, two CNN architectures were introduced in [19]. The first one comprises four convolutional layers, four pooling layers, one fully connected layer, and various intermediary layers for data normalization. The second CNN architecture was intended to extract the visual properties of the images while a Kernel Extreme Learning Machine (KELM) [20] was adopted to address the classification task. Similarly, the network outlined in [21] consists of six layers: a convolutional layer, rectified linear unit (ReLU), and max-pooling layer, respectively. In addition, a total of five dropout layers was used to avoid overfitting. Recently, a deep learning and multi-scale feature-based glioma classification approach was outlined in [22]. Particularly, a fusion technique was designed for refining and combining the visual descriptors extracted at different scales of the network. Similarly, a CNN fusion network with several streams’ inputs was depicted by the researchers in [23]. Each of the three sensors; enhanced-T1-MRI, T2-MRI, and FLAIR, feeds its associated stream of CNN with 2D brain image slices. The three-stream CNN features are then aggregated using a fusion layer. In [24], a CNN-based technique was suggested for categorizing glioma brain tumor MR images. The authors used a genetic algorithm to determine the best-performing CNN structure in classifying the grading of glioma tumors. In [7], the researchers used the CNN’s weights inherited from a pre-trained VGG192 for a block-wise fine-tuning and improving the classification results. In [25], the researchers introduced an AlexNet [26]-based approach to predict the histological grade of canine meningiomas.

The authors in [27] outlined a model intended to determine the firmness of meningiomas using T2-weighted MRI. Specifically, they investigated texture features, including local binary patterns (LBP) [28], the gray level co-occurrence matrix (GLCM) [29], and discrete wavelet transform (DWT) [30]. The extracted features were then used in conjunction with two classifiers; Support Vector Machines (SVM) and k-Nearest Neighbors (KNN), to categorize the regions of interest (ROIs) as either soft or firm. In [6], a supervised machine learning-based CAD was introduced to detect meningioma firmness based on MRI instances. The researchers used distinct feature space regions to build base learners and form a multi-learner ensemble based on possibilistic clustering for the firmness category prediction. In [31], the authors extracted radiomics features from multi-parametric MRI. They used variance selection and least absolute shrinkage and selection operator (LASSO) regression for feature selection. Furthermore, they built radiomics models to classify the meningioma firmness using five classifiers: Random Forest (RF), KNN, the SVM, Logistic Regression (LR), and the AdaBoost Classifier (Ada). The area under curve (AUC) was used to evaluate the performance of each classifier. Similarly, the researchers in [5] explored classification algorithms to predict the consistency of meningiomas. In addition, they used clinical results of ultrasonic elastography and radiometric characteristics of pre-operative MRIs for features encoding. The optimal results were obtained by combining Information Gain and ReliefF feature selection methods, which were then applied to the Naive Bayes classifier. In [32], the researchers utilized the YOLO model [33] to design a deep learning-based approach for brain tumor firmness classification. They evaluated the performance achieved using five YOLO versions. In their study, YOLOv7 demonstrated superior performance compared to the remaining considered versions of the model.

On the other hand, unsupervised deep learning techniques, such as autoencoders [34,35] and Generative Adversarial Networks (GANs) [36,37,38,39] relaxed the need for labeled data. Moreover, they have alleviated the scarcity of labeled medical datasets. In fact, they have been exploited to extract relevant features in an unsupervised manner, and address medical image classification tasks. In particular, two studies utilized stacked denoising autoencoders for unsupervised feature extraction [34,35]. The first one fed the extracted features into a support vector machine classifier [34], while the second study used a logistic regression model to classify noisy hyperspectral images [35]. In addition, the research in [40] showcased an unsupervised generic descriptor extraction from the OverFeat network, and coupled it with a variety of recognition tasks. Consistently, the authors in [36] outlined a pre-trained GAN-based clustering approach designed to extract fine-grained semantic features. In [37], the researchers introduced a Deep Convolutional Generative Adversarial Network (DCGAN) architecture for unsupervised feature extraction. The study aimed at demonstrating empirically the ability of DCGAN to learn an efficient hierarchy of representations from object parts to scenes in both the generator and discriminator. In [38], the DCGAN was employed for spectral-spatial feature extraction from hyperspectral imagery. Furthermore, Wasserstein Generative Adversarial Network (WGAN) [39] mitigated GAN instability by using Wasserstein Distance for feature extraction.

The deep learning approaches outlined above succeeded to relax the need for expert-driven feature engineering and extract discriminative features relevant to the intended classification task [8]. However, such supervised deep learning approaches require labeled datasets often built through manual annotation [41]. Furthermore, the state-of-the-art approaches exhibit relatively high complexity. On the other hand, the Bidirectional Generative Adversarial Networks (BiGANs) [42], represent an unsupervised learning technique that extracts rich feature representations using unlabeled data. Specifically, BiGAN’s framework generates realistic synthetic samples while training simultaneously the encoder to learn powerful feature representations. In addition, the depth-wise separable convolutional layer presents a valuable alternative to learn richer representations and reduce significantly the complexity of the intended model. Accordingly, BiGAN-based feature extraction along with the depth-wise separable convolutional layer represent considerable potential to address challenges relevant to meningioma firmness detection.

In this research, we propose an adversarial feature learning-based meningioma firmness classification approach. Specifically, we introduce two main contributions intended to address the considered MRI-based classification problem. First, a novel unsupervised deep learning-based feature extraction approach is employed to relax the need for handcrafting and/or engineering MRI descriptors. Precisely, the proposed BiGAN encoder learns a set of features that enhances the discriminative ability of the subsequent classification model. Second, a depth-wise separable deep learning model is designed for meningioma firmness classification. The proposed model associates the BiGAN encoder-based features with the depth-wise separable convolutional network to learn richer representations while significantly reducing the model complexity.

## 2. Materials and Methods

This research introduces a novel meningioma firmness classification approach that leverages the features extracted through adversarial feature learning and conveys them into depth-wise separable convolutional layers to learn richer data representations and map them into the predefined meningioma categories.

### 2.1. Adversarial Learning-Based Feature Extraction

**Figure 1:**
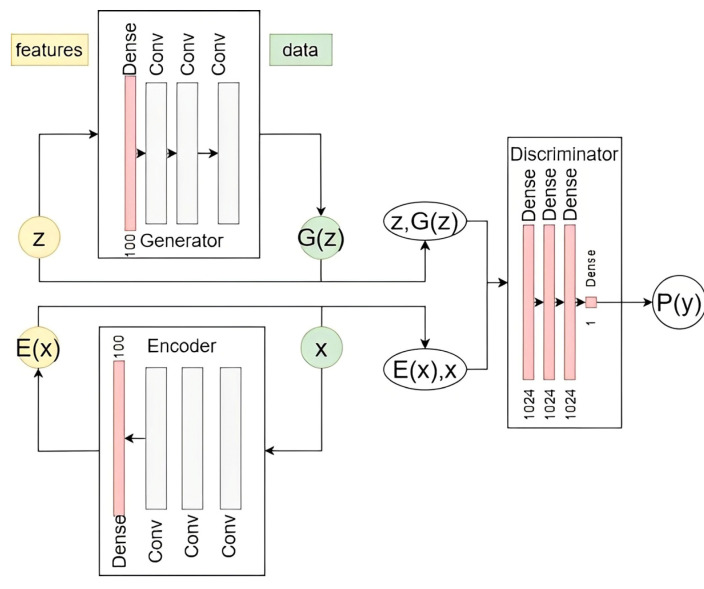
Structure of the proposed Bidirectional Generative Adversaries Network-based feature extraction.

As can be seen in Figure 1, the proposed adversarial learning-based feature extraction consists of three components: (i) a discriminator that differentiates the joint distribution of (x, E(x)) and (G(z), z), (ii) an encoder that maps the data instance x into a latent distribution E(x),  and (iii) a generator that maps the latent distribution z to G(z). One should mention that network E  takes real data samples x and maps them to their latent representations E(x). On the other hand, network G takes a latent input z and generates the corresponding output G(z). In addition, the role of network D consists in discriminating between the input pairs, (G(z), z) and (x, E(x)). It is trained to accurately classify these input pairs, with the goal of identifying the (z, G(z)) pairs as “fake” and the (E(x), x) pairs as “real”.

Specifically, the training of the considered Bidirectional Generative Adversarial Network (BiGAN) relies on the optimization of three loss functions. Namely, the generator loss GL and the encoder loss EL  are minimized, while the discriminator loss DL is maximized. During the training process, these losses are used to update the parameters of the three key networks that make up the BiGAN architecture. In particular, optimizing the parameters of the discriminator network D depends solely on minimizing DL, while GL  and EL are omitted. In fact, the discriminator loss DL penalizes discriminator D for misclassifying x  as fake or classifying the generated fake samples, G(z), as real. Note that GL and EL are designed to penalize G and E, respectively, when D is not “fooled”. In other words, they are intended to improve G and E performance to the point where D can no longer reliably differentiate the generated/encoded samples from the real records. This interplay between D, G, and E is a fundamental aspect of the adversarial training process that underlies the BiGAN model’s ability to learn powerful data representations. The objective function for the proposed BiGAN model can be described as follows:

(1) $$\min_{\left(G,E\right)}\max_{D}\left(D,E,G\right)=E_{x\sim p_{X}(x)}E_{z\sim P_{E}(z|x)}log[D(x,E(x)]+E_{z\sim p_{Z}\left(z\right)}E_{x\sim P_{G}\left(x|z\right)}log[1-D(G(z),z)]$$

where $E_{x\sim p_{X}(x)}$ and $E_{z\sim p_{Z}\left(z\right)}$ denote the probability of real and fake distributions. $P_{E}(z|x)$ is a probability distribution where the encoder E  takes x as input and produces z. Conversely, $P_{G}\left(x|z\right)$ represents the probability distribution where the generator G takes x as input and produces z. In addition, $D(x,E\left(x\right))$ is a discriminator output given x and $E\left(x\right)$, which is the output of the encoder E given the real input x. Lastly, D(G(z),z) represents a discriminator output given z  and G(z), which is the output of the G given the input z.

In this research, the BiGAN encoder includes, first, a convolutional layer with 32 filters of size 3×3. Next, a LeakyReLU activation function with a small negative slope (alpha=0.2) is applied to the convolutional layer output. One should note that this leaky ReLU function addresses the dying ReLU problem and improves the gradient flow within the network. Moreover, it leads to a more resilient and stable training. Specifically, rather than completely discarding negative inputs, the leaky ReLU incorporates a slight slope or leakage for the negative values [43]. Then, a dropout layer is placed to randomly set 25% of the input units to zero during each training iteration. This introduces randomness by selectively removing units and their connections from the neural network during the training to effectively mitigate overfitting. The next convolutional layer encloses 64 filters of size 3×3. The subsequent layer is a BatchNormalization layer, that subtracts the mean and divides by the batch’s standard deviation to normalize the activations of the preceding layer. The latter layer is intended to significantly speed up the training, and enable the utilization of higher learning rates, leading to better accuracy, and reducing the need for precise weight initialization. The output of the BatchNormalization layer is then passed through a leaky ReLU activation function and a dropout layer. The third convolutional layer includes 128 3×3-sized filters. This is followed by a BatchNormalization layer, a leaky ReLU activation function, and a dropout layer, respectively. Finally, the flattened output is fed into one dense layer with 100 neurons.

The features extracted using the BiGAN encoder detailed above are then fed into the subsequent downstream model. In particular, this study investigated a basic BiGAN model as well as a pre-trained BiGAN to implement the proposed encoder. Moreover, the detailed structure of the MobileNet model used for the classification task is revealed in Table 1. As it can be seen, we provide the layer name, the input shape, the filter size, the stride and padding details, and the output shape associated with each MobileNet layer. Note that the input image size is 56×56. The layers named “conv_dw_1” and “conv_pw_1” refer to the first depth-wise convolutional layer and the first point-wise convolutional layer, respectively. An important aspect to mention is that the weights of the layers located between “conv1” and “conv_dw_4” were frozen during the training process. This means that the parameters of those layers were not updated when building the model. In contrast, the layers located between “conv_pw_4” and “conv_pw_13” were fine-tuned and updated during the training. Consequently, the extracted features were conveyed to the depth-wise separable convolutional layers for further processing.

The discriminator module of the deployed BiGAN model, shown in Figure 1, encloses three fully connected (dense) layers, each with 1024 neurons. In addition, a leaky ReLU activation function and a dropout layer are placed next. Moreover, the architecture includes one additional dense layer with a single neuron that is dedicated to the classification task. On the other hand, the generator begins with a dense layer, which serves as input to the model. The size of the latter dense layer is set to 100, representing the size of the receptive field. Following the dense input layer, the generator employs two convolutional layers. The first one includes 128 filters, each of size 3×3, while the second convolutional layer has 64 filters, also of size 3×3. Also, the generator architecture incorporates BatchNormalization layers after each convolutional layer. Furthermore, the generator utilizes (rectified linear unit) ReLU activation functions in conjunction with the BatchNormalization layers. ReLU is used to introduce non-linearity and mitigate the vanishing gradient problem. Finally, the generator architecture includes up-sampling layers after each BatchNormalization and ReLU block. These up-sampling layers are responsible for extracting and combining the features collected from the input tensor. The last layer consists of a convolutional layer including 3  filters of size 3×3, followed by a hyperbolic tangent (Tanh) activation function. In fact, the Tanh function is typically employed to achieve specific image normalizations in the generator. Consequently, the generated output images’ pixel values are squashed within the range of [−1, 1].

### 2.2. Depth-Wise Separable Deep Learning

**Figure 2:**
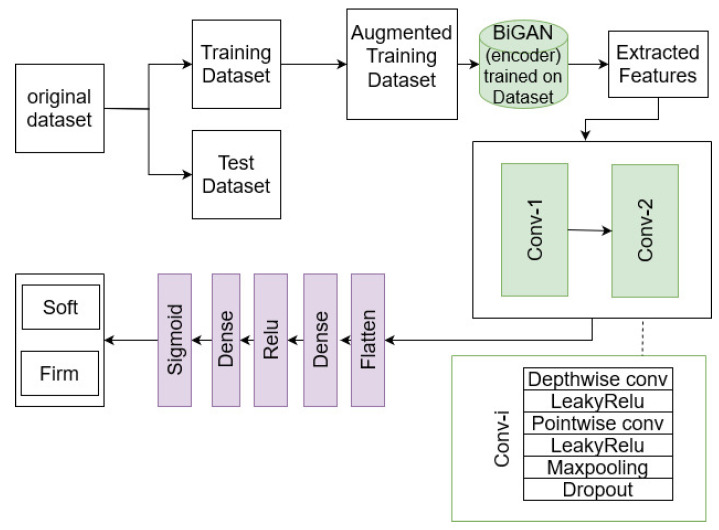
Proposed approach for meningioma firmness detection.

As illustrated in Figure 2, the structure of the supervised deep learning model includes depth-wise separable convolutional layers. This aims at reducing the risk of overfitting and reducing the number of model parameters compared to typical CNN architectures that rely on regular convolutional layers with a fixed network depth. In fact, the depth-wise separable convolution employed in the proposed network consists of a depth-wise convolutional layer followed by a point-wise convolutional layer that splits the convolutional operation into a 3×3 depth-wise convolution and a 1×1 point-wise convolution. The rationale behind adopting this depth-wise separable convolutional architecture is its ability to capture the important fine details and structures, such as small tumors or lesions, present in the brain MRI. As one can see, the core of the architecture includes two “conv-i” blocks, each of which employs separable convolutional operations as a combination of depth-wise and point-wise convolutions. The first block utilizes a separable convolutional layer with 128 filters with a size of 7×7, while the second layer employs 32 filters with a size of 3×3. These convolutional operations are activated using the ReLU function. Following the convolutional layers, a max-pooling operation with a 2×2 filter is applied to each conv-i block.

Additionally, the model incorporates a dropout layer with a rate of 0.5, which randomly disables some of the neuron activations. Next, the feature maps are flattened and passed through two dense (fully connected) layers: the first containing 256 neurons and the second, 1 neuron. The first dense layer uses a ReLU activation, while the second employs a sigmoid activation function. The sigmoid function is particularly well-suited for binary classification tasks, as it maps the input values to the range of [0,1], providing a probabilistic interpretation of the network’s output. This probabilistic output can be interpreted as the likelihood of the input belonging to a specific class [44]. Finally, the output layer of the network is responsible for mapping the extracted features into the pre-defined class labels.

## 3. Experiments

**Figure 3:**
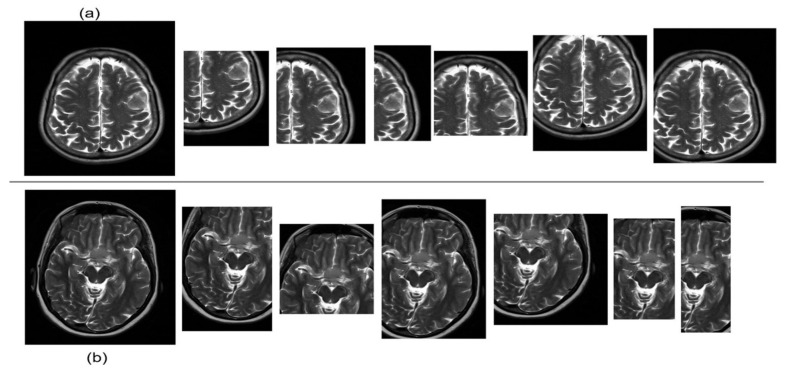
Sample data cropping, including 6 cropped images for (**a**) firm and (**b**) soft cases.

In the experiments, a collection of brain tumor MRIs was provided by King Khalid University Hospital (KKUH) in Riyadh, Saudi Arabia. This dataset was collected from 28 patients with meningioma, comprising nineteen firm cases and nine soft cases which were assessed by a medical expert. The images were cropped and automatically labelled, yielding a total of 837 ground truth images. It should be noted that the dataset was further augmented, yielding a total of 4139 records. The resulting class distribution remained at 32% for soft meningioma and 68% for firm meningioma. Figure 3 shows examples of firm and soft MRIs, along with the corresponding six cropped images. The preprocessing steps used to prepare the data for the experiments include image resizing, which modifies the dimensions (width and height) of the images to a desired size. The training data were augmented using random horizontal flipping and small random rotations within a range of ±0.2 radians.

For the validation and assessment of the proposed approach, the Adam optimizer was associated with a learning rate of 0.0001 and a momentum value of 0.5. Moreover, different batch sizes were investigated: thirty-two for the basic BiGAN, sixty-four for the pre-trained BiGAN, and eight for the depth-wise separable deep learning model. Furthermore, the training was performed using a binary cross-entropy loss function. In addition, a label smoothing operation was deployed to mitigate the risk of overfitting. This involved the introduction of soft labels as ground-truth labels of the training data. Such label smoothing penalizes overly confident predictions by adding a degree of uncertainty to the labels, thereby encouraging the model to learn more nuanced and generalized representations.

**Figure 4:**
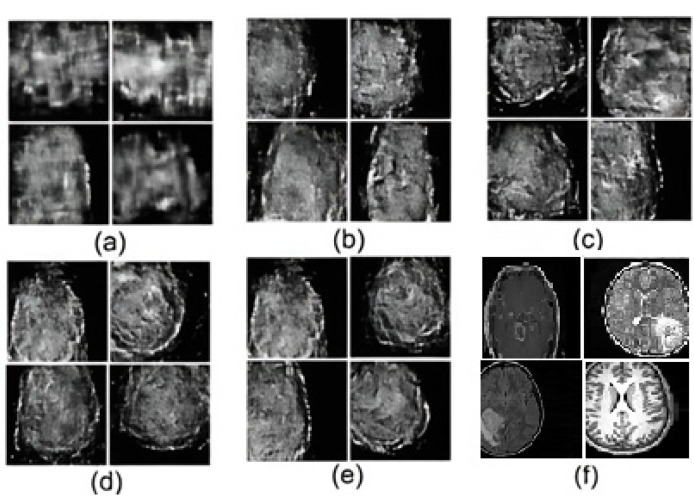
Sample images generated using a (**a**) pre-trained BiGAN at epoch 15 k, (**b**) pre-trained BiGAN at epoch 46 k, (**c**) pre-trained BiGAN at epoch 54 k, (**d**) pre-trained BiGAN at epoch 76 k, (**e**) pre-trained BiGAN at epoch 88 k, and (**f**) pre-trained BiGAN at epoch 120 k.

First, we showcased sample MRI images generated by a pre-trained MobileNet-based BiGAN. As can be seen in Figure 4, sample images were generated at epochs 15 k, 46 k, 54 k, 76 k, 88 k, and 120 k, respectively. The generated images showcase the ability of the proposed BiGAN-based feature extraction to learn relevant meningioma descriptors. Further investigation revealed that the generated images exhibit improved details starting from epoch 76 k. This suggests the model had learned progressively more relevant features from the MRI data. Moreover, it is important to note that the instances generated by the pre-trained BiGAN at epochs 88 k and on exhibit a better separation between brain tissue and the surrounding area. Furthermore, starting approximately from epoch 120 k, sharper and more refined descriptors can be seen. In other words, subtler nuances and intricate structures are captured by the model. This confirms the model’s ability to extract relevant descriptors from the considered MRI images.

**Figure 5:**
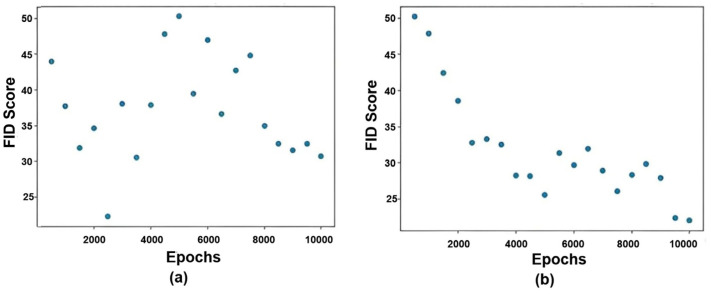
FID scores recorded using (**a**) basic BiGAN and (**b**) pre-trained BiGAN.

The Fréchet Inception Distance (FID) score [45] was also employed to evaluate the quality of the generative MRI. As can be seen in Figure 5, FID scores were calculated at various epochs while training basic and pre-trained BiGAN models. For the basic BiGAN model, FID scores were recorded every 500 epochs, and a fluctuation was observed until the $8000^{th}$ epoch. This reflects the instability of the generated images’ quality. Then, the FID scores start decreasing which proves that the model is generating more realistic images. In the case of the pre-trained BiGAN model, FID scores were recorded every 5000 epochs. The obtained results show a gradual decrease until the ${100,000}^{th}$ epoch. This indicates that the model is gradually succeeding in generating more realistic images as the training progresses.

While training a BiGAN, we adjusted the hyperparameters to ensure optimum training stability. These hyperparameters include the cross-entropy smoothing factor, the batch size, as well as the number of the discriminator’s layers. Particularly, we experimented with the values of 0.1 and 0.3 for the cross-entropy smoothing factor. On the other hand, the values four, eight, sixteen, thirty-two, sixty-four, and one hundred twenty-eight were considered for the batch size. In addition, the number of discriminator layers was set to two, three, five, and seven.

**Figure 6:**
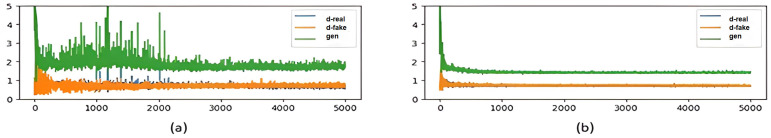
Comparison of BiGAN losses curve during training using binary cross-entropy smoothing values of (**a**) 0.1 and (**b**) 0.3.

Figure 6 shows the BiGAN losses curves recorded for training the model with different values of binary cross-entropy smoothing values. As it can be seen, smoother values, such as 0.3, led to a more stable training process and lower loss values. However, a larger smooth value leads to less discrimination between real and fake samples. Accordingly, for remaining experiments, we set the smoother value to 0.3.

**Figure 7:**
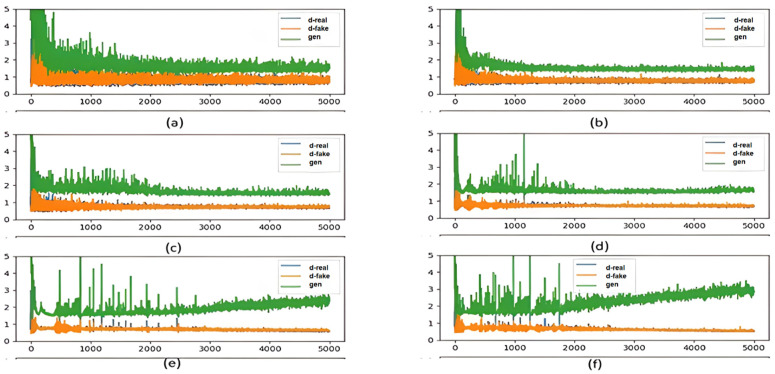
The generator loss and discriminator loss over the 5000 epochs while adjusting the batch size to (**a**) four, (**b**) eight, (**c**) sixteen, (**d**) thirty-two, (**e**) sixty-four, and (**f**) one hundred twenty-eight.

As depicted in Figure 7, setting the batch size to 16 and 32 yielded more stable updates of the encoder, generator, and the discriminator weights. In fact, the gradients were averaged over more samples, leading to a smoother loss curve and better overall performance. As one can see, using larger batch sizes of 64 and 128 made the generator loss unstable and increasing over time. Moreover, the larger batch size made the discriminator overfit the training data and affected the model generalization capability.

**Figure 8:**
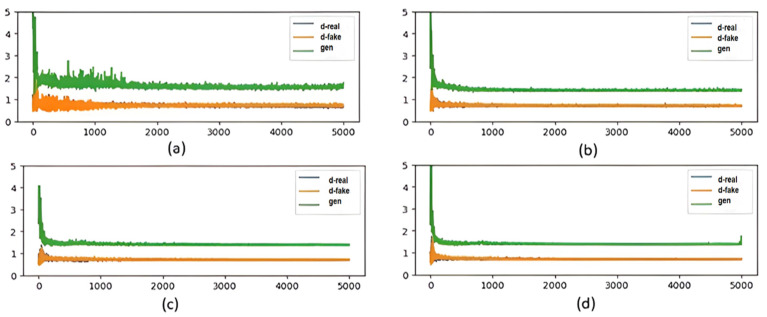
The generator loss and discriminator loss over the 5000 epochs with discriminators having (**a**) two layers, (**b**) three layers, (**c**) five layers, and (**d**) seven layers.

Furthermore, we investigated the impact of the discriminator complexity on the performance of BiGAN as reported in Figure 8. Specifically, the BiGAN model was trained with discriminators enclosing different numbers of dense layers ranging from two to seven. The discriminator, generator, and encoder losses were monitored during the model training. The results showed that increasing the discriminator’s complexity from two to three dense layers improved the stability of BiGAN because the discriminator and generator losses decreased and became more stabilized. This suggests that the discriminator, encoder, and generator all stabilize over time. However, increasing the complexity of the discriminator beyond three dense layers did not yield significant performance gains. Specifically, adding more layers, such as five or seven, helped stabilize the losses to some extent, but the impact was limited. Therefore, we chose to implement three layers in our proposed approach to achieve a balance between stability and efficiency.

**Figure 9:**
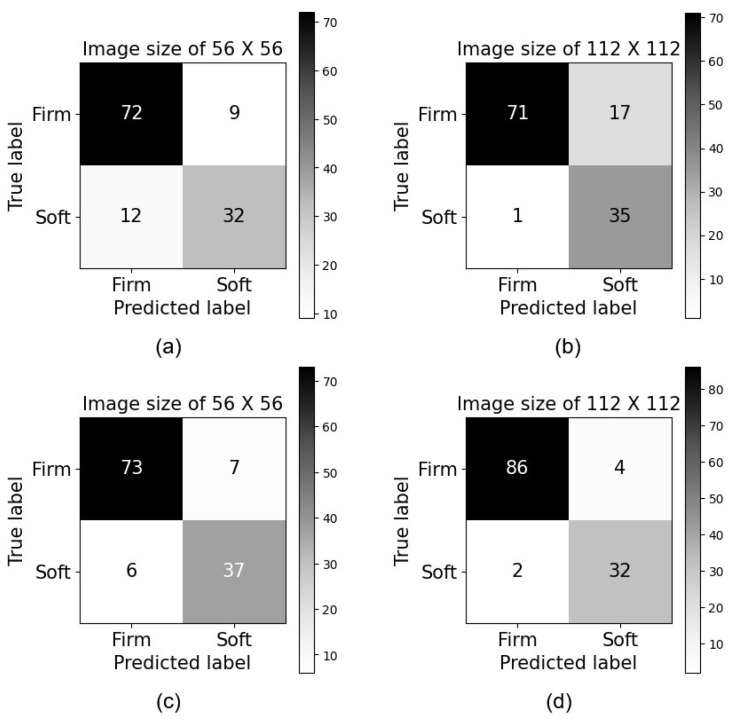
Confusion matrix obtained using the basic BiGAN model and images of size (**a**) 56 × 56 and (**b**) 112 × 112, and pre-trained BiGAN model and images of size (**c**) 56 × 56 and (**d**) 112 × 112.

Figure 9 reports the arithmetic means computed using the confusion matrices obtained using the five-fold cross-validation. These results represent the classification performance of two deep learning models: the basic BiGAN model and the pre-trained BiGAN model. Both models utilized BiGAN encoders for feature extraction in the classification task. In Figure 9b, the confusion matrix obtained using the basic BiGAN model and 112×112 images shows slightly better results for the soft class compared to the results achieved by the basic BiGAN model and 56×56 images shown in Figure 9a.

On the other hand, the confusion matrix obtained using the pre-trained BiGAN model with 112×112 images shown in Figure 9d reflects an improved classification performance compared to the pre-trained BiGAN model results obtained using 56×56 images shown in Figure 9c. The confusion matrices for the pre-trained BiGAN models shown in Figure 9c,d report better results compared to the performance of basic BiGAN models shown in Figure 9a,b. This implies that the pre-trained models have learned more representative features and can better discriminate between classes. Based on the obtained results, one can claim that using larger image sizes of 112×112 yields improved classification accuracy for both basic and pre-trained BiGAN models. Additionally, the pre-trained models outperform the basic models in terms of classification accuracy.

Additionally, Table 2 reports the classification performances obtained using the considered five-folds cross-validation along with the corresponding standard deviation. Namely, the metrics included in the comparison are accuracy, precision, recall, weighted F1-score, and Cohen’s kappa coefficient.

The comparison explores relevant unsupervised feature extraction approaches as well as depth-wise separable deep learning models. Particularly, the first four related works rely on well-established deep learning architectures, namely NASNetLarge [46], DenseNet201 [47], ResNet50 [48], and VGG16 [49] as feature extraction backbones. These models were used to obtain meaningful representations of the input images. On the other hand, the work in [50] encloses an autoencoder-based unsupervised feature extraction approach. In fact, the autoencoder was intended to reconstruct the conveyed input, thereby capturing the most salient features in an unsupervised manner. Regardless of the unsupervised feature extraction technique used—whether it employed an autoencoder or well-established deep learning architecture networks—the extracted features were then fed into depth-wise separable deep learning models to achieve the classification task. The related work in [27], represents a supervised machine learning-based CAD system that automatically classifies meningiomas as soft or firm. It exploits LBP as a feature extraction technique and couples it with an ensemble learner to categorize meningioma cases. Namely, KNN, hard SVM, and RBF-SVM models were combined to form the ensemble learner. Both DCGAN [38] and WGAN [39] are generative adversarial network architectures typically used for feature extraction. Specifically, the extracted features were subsequently conveyed to a depth-wise separable deep learning model to achieve the intended classification task.

The results obtained using the latter state-of-the-art approaches are compared with those achieved by the proposed (i) depth-wise separable deep learning-based, (ii) basic BiGAN-based, and (iii) pre-trained BiGAN-based approaches. Note that the extracted BiGAN-based features were then fed as input into the depth-wise separable deep learning model. The results reported in Table 2 show that the proposed pre-trained BiGAN-based model yielded the highest results among all of the models. This performance can be attributed to the transfer learning capability to extract discriminative features for meningioma firmness classification based on the knowledge and representations learned through extensive pre-training using larger datasets. On the other hand, the depth-wise separable deep learning model, which was not preceded by an unsupervised feature extraction, yielded the least promising results. The basic BiGAN-based approach yielded higher accuracy, weighted F1-score, and Cohen’s kappa coefficient compared to the performance of the depth-wise separable deep learning model. Specifically, the BiGAN-based model increased the accuracy, the weighted F1-score, and Cohen’s kappa coefficient from 72.32 to 87.52, from 0.73 to 0.87, and from 0.44 to 0.72, respectively. This performance can be attributed to the BiGAN encoder’s capability to extract more discriminative features, which improved its overall performance. As can be seen, the considered basic BiGAN-based model outperformed the autoencoder-based approach. It is also worth noting that BiGAN’s adversarial training yielded more powerful and discriminative data representations. This translated to superior performance on downstream tasks compared to autoencoder-based features. This also demonstrates BiGAN’s ability to generate authentic synthetic samples while concurrently training the encoder to acquire robust feature representations. In addition, using NASNetLarge, DenseNet201, ResNet50, and VGG16 models as pre-trained models for unsupervised feature extraction without fine-tuning constrained the learning of nuanced task-specific features and yielded lower performance. In particular, the ResNet50 model achieved the lowest performance. On the other hand, the proposed pre-trained BiGAN-based and the basic BiGAN-based approaches showcased better performance than the work in [27]. This arises from the deep learning algorithms’ ability to automatically mine complex patterns from data. Moreover, the proposed pre-trained BiGAN model achieved a recall of 0.87 (+/−0.14) and outperformed the other models. The latter pre-trained BiGAN-based model yielded the highest precision, indicating the best ability to correctly identify positive cases out of all of the predicted positive cases. A comparative analysis of BiGAN and GAN-based models demonstrates that BiGAN models outperform GAN-based models, achieving higher weighted F1 scores, recall, and Cohen’s kappa coefficients.

Given the results reported in Table 2, one can notice that the considered models exhibit varying levels of agreement as measured by their Cohen’s kappa coefficients (κ). Particularly, the depth-wise separable deep learning model and the work in [27] yielded a moderate level of agreement, with kappa coefficients falling in the range of 0.41 ≤ κ ≤ 0.60. On the other hand, NASNetLarge, DenseNet201, VGG16 and Autoencoder-based models, DCGAN, and WGAN, along with the proposed basic BiGAN-based model demonstrated a good level of agreement, with kappa coefficients within the range of 0.61 ≤ κ ≤ 0.80. Notably, the proposed pre-trained BiGAN-based model stood out with the highest kappa coefficient reaching 0.86 (+/−0.10) and indicating a substantial to very good level of agreement. Furthermore, Table 3 reports the execution time spent by the considered deep learning-based models. As one can see, the VGG16-based approach required a longer execution time of 168.34 s. In contrast, the ResNet50-based solution took a shorter execution time of 15.94 s with a low Weighted F1-score of 0.70 (+/−0.05). On the other hand, the proposed basic BiGAN-based approach strikes a balance, achieving a weighted F1-score of 0.87 (+/−0.02) with a shorter execution time of 15.38 s. This indicates that the features extracted using the BiGAN helped decrease the inference time of the proposed models, making them better suited for real-world applications with tighter time or resource constraints.

**Figure 10:**
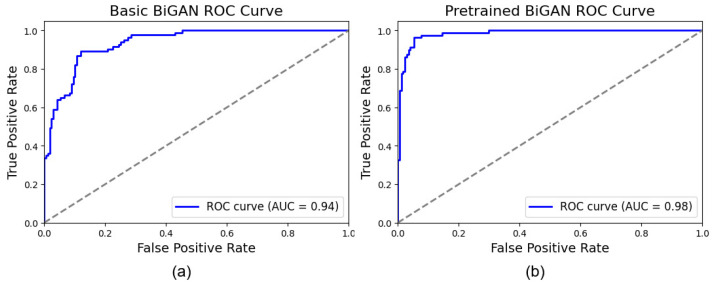
Receiver operating characteristic (ROC) curves: (**a**) basic BiGAN and (**b**) pre-trained BiGAN.

Figure 10 shows the receiver operating characteristic (ROC) curves, which illustrate the trade-off between true positive and false positive rates for the considered BiGAN-based models. As can be seen, the basic BiGAN-based model achieved an area under curve (AUC) of 0.94, indicating a considerable ability to distinguish between the pre-defined classes. Consistently, the pre-trained BiGAN-based model yielded a superior AUC score of 0.98 suggesting an enhanced classification performance. This confirms that associating the MobileNet model with the pre-trained BiGAN-based model enhances the discrimination capability of the proposed approach.

## 4. Conclusions

Meningioma firmness classification through the association of image processing techniques with radiologists’ visual assessments of MRIs proved to be time-consuming and subjective to the physician’s judgment. On the other hand, the earliest machine learning-based solutions relied on hand-crafted attributes and/or feature engineering techniques to encode the content of patient imaging modalities. This article introduced a novel deep learning approach, designed and implemented to address the meningioma firmness classification problem. Specifically, an unsupervised feature extraction based on Bidirectional Generative Adversary Networks was proposed to determine discriminative features relevant to meningioma firmness classification. Moreover, a depth-wise separable learning architecture was designed to map the learned features into the pre-defined meningioma firmness classes. These research contributions were validated using a real dataset including MRIs along with relevant performance measures. Moreover, the performance of the proposed approach was compared with the results obtained using relevant state-of-the-art techniques. The experiments demonstrated the BiGAN encoder’s capability to extract relevant features in an unsupervised manner. In addition, the considered deep learning model yielded better firmness detection performance and outperformed existing models. Specifically, it achieved an accuracy of 94.7% and a weighted F1-score of 95.0%. This demonstrated the proposed model’s ability to extract discriminative features and accurately classify meningioma consistency. The insights gained from this research highlight the importance of unsupervised feature extraction in cancer classification tasks. They also reduced the reliance on labeled data and enabled the discovery of latent patterns and representations.

As a future work, other pre-trained models can be investigated for meningioma firmness classification. Moreover, ensemble learning might improve further base learners’ performance. Furthermore, the self-supervised deep learning paradigm can also be studied to alleviate data scarcity.

## Figures and Tables

**Table 1:** Point-wise convolutional and depth-wise convolutional layers of the considered MobileNet model.

Layer Name	Input Shape	Filter Size	Stride	Padding	Output Shape
conv2d	(56, 56, 3)	(3, 3)	(2, 2)	same	(28, 28, 32)
conv_dw_1	(28, 28, 32)	(3, 3)	(1, 1)	same	(28, 28, 32)
conv_pw_1	(28, 28, 32)	(1, 1)	(1, 1)	same	(28, 28, 64)
conv_dw_2	(29, 29, 64)	(3, 3)	(2, 2)	valid	(14, 14, 64)
conv_pw_2	(14, 14, 64)	(1, 1)	(1, 1)	same	(14, 14, 128)
conv_dw_3	(14, 14, 128)	(3, 3)	(1, 1)	same	(14, 14, 128)
conv_pw_3	(14, 14, 128)	(1, 1)	(1, 1)	same	(14, 14, 128)
conv_dw_4	(15, 15, 128)	(3, 3)	(2, 2)	valid	(7, 7, 128)
conv_pw_4	(7, 7, 128)	(1, 1)	(1, 1)	same	(7, 7, 256)
conv_dw_5	(7, 7, 256)	(3, 3)	(1, 1)	same	(7, 7, 256)
conv_pw_5	(7, 7, 256)	(1, 1)	(1, 1)	same	(7, 7, 256)
conv_dw_6	(8, 8, 256)	(3, 3)	(2, 2)	valid	(3, 3, 256)
conv_pw_6	(3, 3, 256)	(1, 1)	(1, 1)	same	(3, 3, 512)
conv_dw_7	(3, 3, 512)	(3, 3)	(1, 1)	same	(3, 3, 512)
conv_pw_7	(3, 3, 512)	(1, 1)	(1, 1)	same	(3, 3, 512)
conv_dw_8	(3, 3, 512)	(3, 3)	(1, 1)	same	(3, 3, 512)
conv_pw_8	(3, 3, 512)	(1, 1)	(1, 1)	same	(3, 3, 512)
conv_dw_9	(3, 3, 512)	(3, 3)	(1, 1)	same	(3, 3, 512)
conv_pw_9	(3, 3, 512)	(1, 1)	(1, 1)	same	(3, 3, 512)
conv_dw_10	(3, 3, 512)	(3, 3)	(1, 1)	same	(3, 3, 512)
conv_pw_10	(3, 3, 512)	(1, 1)	(1, 1)	same	(3, 3, 512)
conv_dw_11	(3, 3, 512)	(3, 3)	(1, 1)	same	(3, 3, 512)
conv_pw_11	(3, 3, 512)	(1, 1)	(1, 1)	same	(3, 3, 512)
conv_dw_12	(4, 4, 512)	(3, 3)	(2, 2)	valid	(1, 1, 512)
conv_pw_12	(1, 1, 512)	(1, 1)	(1, 1)	same	(1, 1, 1024)
conv_dw_13	(1, 1, 1024)	(3, 3)	(1, 1)	same	(1, 1, 1024)
conv_pw_13	(1, 1, 1024)	(1, 1)	(1, 1)	same	(1, 1, 1024)

**Table 2:** The classification performance achieved by the related works and the proposed models.

Approach	Accuracy	Precision	Recall	Weighted F1_Score	Cohen’s Kappa Coefficient
NASNetLarge [46]	86.29 (+/−1.12)	0.72 (+/−0.04)	0.86 (+/−0.09)	0.87 (+/−0.01)	0.68 (+/−0.02)
DenseNet201 [47]	86.72 (+/−3.14)	0.93 (+/−0.04)	0.65 (+/−0.10)	0.86 (+/−0.04)	0.68 (+/−0.09)
ResNet50 [48]	74.56 (+/−0.93)	0.43 (+/−0.22)	0.46 (+/−0.13)	0.70 (+/−0.05)	0.19 (+/−0.13)
VGG16 [49]	88.16 (+/−2.17)	0.85 (+/−0.07)	0.82 (+/−0.08)	0.88 (+/−0.02)	0.74 (+/−0.05)
Autoencoder [50]	84.80 (+/−1.82)	0.81 (+/−0.06)	0.79 (+/−0.08)	0.85 (+/−0.02)	0.67 (+/−0.04)
The approach in [27]	80.0 (+/−0.01)	0.77 (+/−0.02)	0.54 (+/−0.04)	0.79 (+/−0.01)	0.50 (+/−0.03)
DCGAN [38]	86.37 (+/−2.49)	0.87 (+/−0.05)	0.75 (+/−0.12)	0.86 (+/−0.03)	0.70 (+/−0.07)
WGAN [39]	85.34 (+/−2.16)	0.82 (+/−0.04)	0.76 (+/−0.05)	0.85 (+/−0.02)	0.67 (+/−0.05)
Depth-wise separable deep learning-based approach	72.32 (+/−2.18)	0.56 (+/−0.03)	0.80 (+/−0.15)	0.73 (+/−0.02)	0.44 (+/−0.05)
Basic BiGAN-based approach	87.52 (+/−1.30)	0.87 (+/−0.09)	0.80 (+/−0.12)	0.87 (+/−0.02)	0.72 (+/−0.04)
Pre-trained BiGAN-based approach	94.72 (+/−3.34)	0.94 (+/−0.06)	0.87 (+/−0.14)	0.95 (+/−0.04)	0.86 (+/−0.10)

**Table 3:** The execution time recorded for the related works and proposed models.

Model	Time (s)
NASNetLarge [46]	33.59
DenseNet201 [47]	10.77
ResNet50 [48]	15.94
VGG16 [49]	168.34
Autoencoder [50]	18.04
DCGAN [38]	37.53
WGAN [39]	35.9
Depth-wise separable deep learning-based approach	78.87
Basic BiGAN-based approach	15.38
Pre-trained BiGAN-based approach	19.08

## Data Availability

Data are contained within the article.
